# Measuring temporal patterns in ecology: The case of mast seeding

**DOI:** 10.1002/ece3.7291

**Published:** 2021-03-11

**Authors:** Marcos Fernández‐Martínez, Josep Peñuelas

**Affiliations:** ^1^ PLECO (Plants and Ecosystems) Department of Biology University of Antwerp Wilrijk Belgium; ^2^ CSIC Global Ecology Unit CREAF‐CSIC‐UAB Bellaterra Barcelona Spain; ^3^ CREAF Bellaterra Barcelona Spain

**Keywords:** disparity, frequency, reproduction, seeds, stability, synchrony, variability

## Abstract

1. Properly assessing temporal patterns is a central issue in ecology in order to understand ecosystem processes and their mechanisms. Mast seeding has traditionally been described as a reproductive behavior consisting of highly variable and synchronized reproductive events. The most common metric used to measure temporal variability and thus infer masting behavior, the coefficient of variation (CV), however, has been repeatedly suggested to improperly estimate temporal variability. Biases of CV estimates are especially problematic for non‐normally distributed data and/or data sets with a high number of zeros.
2. Some recent studies have already adopted new metrics to measure temporal variability, but most continue to use CV. This controversy has started a strong debate about what metrics to use.
3. We here summarize the problems of CV when assessing temporal variability, particularly across data sets containing a large number of zeros, and highlight the benefits of using other metrics of temporal variability, such as proportional variability (PV) and consecutive disparity (*D*). We also suggest a new way to look at reproductive behavior, by separating temporal variability from frequency of reproduction, to allow better comparison of data sets with different characteristics.
4. We suggest future studies to properly describe the temporal patterns in fully scientific and measurable terms that do not lead to confusion, such as variability and frequency of reproduction, using robust and fully comparable metrics.

## Graphical abstract

Temporal variability is one of the foundations of masting literature and has been traditionally measured using the coefficient of variation (CV). However, the use of CV is concerning because of several mathematical drawbacks. We here aim to open a debate on what metrics we should use when dealing with temporal variability in seed production and on whether we should redefine the concept of masting or simply avoid it

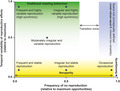

## 1 INTRODUCTION

How to accurately describe temporal patterns in ecological processes has been a long‐standing question in ecology (McCann, 2000). Metrics to estimate stability, temporal variability, and resilience are, actually, still under intense debate (Arnoldi et al., 2016). The field of *masting*, for which assessing temporal patterns of seed production is crucial, is not an exception. Mast seeding, or masting, has traditionally been described as a reproductive phenomenon consisting of the highly variable and synchronized production of seeds at the population scale (Kelly, 1994; Pearse et al., 2016; Silvertown, 1980). The use of the term “masting,” or describing a species or population as following a masting behavior, however, is sometimes confusing, arbitrary, or insufficiently accurate in the scientific literature to be fully accepted as a scientific term (Kelly, 1994). One study even suggested that perpetuating the concept of masting in the scientific literature had no objective basis (Herrera et al., 1998). The concept has nonetheless recently received much attention from the scientific community leading to many new papers, focused on many different aspects and especially on why highly variable reproduction occurs (Ascoli et al., 2017; Bogdziewicz, Ascoli, et al., 2020; Pearse et al., 2017; Vacchiano et al., 2018; Wion et al., 2019), because of the multiple consequences that highly variable reproduction produce in ecosystems, such as cascading effects on food webs (Bogdziewicz et al., 2016; Clotfelter et al., 2007). Clarification of what should be called masting and what should not, however, has received little attention. More important, though, is how we measure the reproductive behavior of organisms (plants or others) independent of arbitrary considerations of them being masting or nonmasting species. To fulfill that aim, we need robust metrics that allow adequate comparisons among species, populations, and individuals. Those metrics still remain under intensive debate in the field of masting, a debate from which many other fields could benefit. Based on previous literature on temporal variability metrics, we here provide some discussion and insights for analyzing time series of reproductive efforts that may help the field establish new and standardized protocols to study and properly measure the reproductive behavior of organisms.

## 2 MEASURING TEMPORAL REPRODUCTIVE BEHAVIOR

**FIGURE 1:**
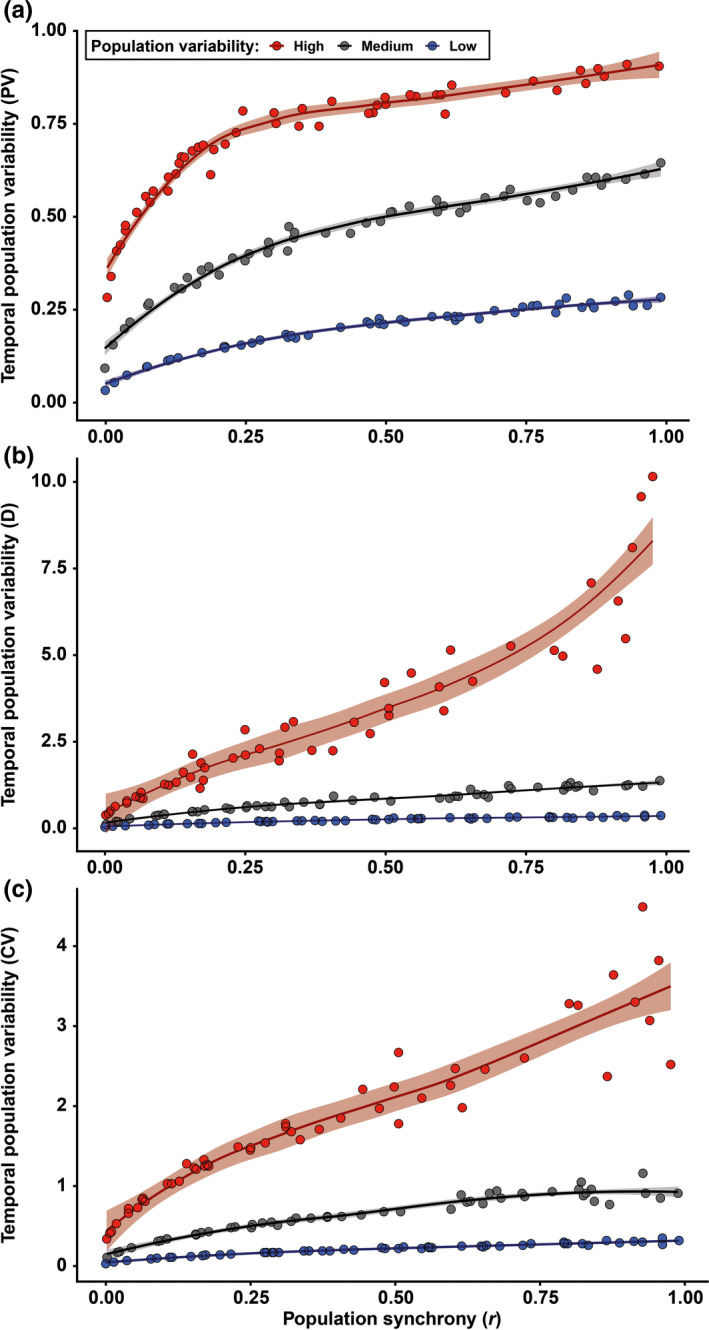
Temporal variability and synchrony among individuals within a population. Relationship between the temporal variability of reproduction (100 years) at the population level as a function of the synchrony among individuals (500) for three levels of variability. Panels (a), (b), and (c) show results using the proportional variability (PV), consecutive disparity (*D*), and coefficient of variation (CV) indices, respectively. The shaded area represents the standard error of the regression line. The code for performing the simulation is available in Supplementary Materials

Three main features have traditionally been used to describe masting: (a) temporal variability of reproductive events (at different levels of organization, e.g., population, individual) most commonly measured using the coefficient of variation (CV = standard deviation · mean^−1^) (Kelly & Sork, 2002), (b) synchrony, often calculated among individuals within a population as the average Pearson's or Spearman's correlation coefficient among all pairwise comparisons (Koenig & Knops, 2013), and (c) temporal autocorrelation, measured with autocorrelation coefficients, as an indication of potential resource depletion (correlation of each value with the previous, lag 1) (Fernández‐Martínez et al., 2019; Pearse et al., 2016) or internal cycles of reproduction or resource mobilization (lags > 1) (Fernández‐Martínez et al., 2012; Sork et al., 1993). The intrinsic link between the temporal variability of reproductive efforts of a population and the average synchrony in reproduction in a population (Buonaccorsi et al., 2003; Herrera, 1998), however, is often overlooked (Figure 1). A strong synchrony among individuals is thus required for a population to have highly variable reproduction, because temporal asynchrony among individuals would lead to a more constant reproduction over a population. This mechanism is actually one of the foundations of the positive relationship between diversity, productivity, and stability in ecosystems (Cardinale et al., 2012; Tilman & Downing, 1994). While high synchrony in reproduction can be found in populations with high and low temporal variability, in a hypothetical stationary state, highly variable reproduction at the population level can only occur at high levels of synchrony. This mathematical property has implications for the traditional definition of masting; “highly variable and synchronous reproduction at the population level” is therefore a redundant definition to some degree. Analyzing temporal variability and synchrony separately can nonetheless still provide relevant information, which is not under debate. Using temporal autocorrelation to study reproductive behavior is also not controversial. How to measure temporal variability, which is the most widely used feature for studying the causes and consequences of reproductive behavior of plants, is currently strongly debated.

## 3 TEMPORAL VARIABILITY: WHY WE NEED ALTERNATIVE MEASURES TO CV

The use of CV as a measure of temporal variability has been debated for decades in various fields of science and even in masting literature itself (Crone et al., 2011; Fernández‐Martínez et al., 2018; Heath, 2006; Martín‐Vide, 1986; McArdle & Gaston, 1995; Mcardle et al., 1990). Computer simulations and heterogeneous data sets have recently been used to test the response of CV under different conditions (Fernández‐Martínez et al., 2018; Heath, 2006; Heath & Borowski, 2013). The results supported previous concerns about the adequacy of using CVs to assess differences in temporal variability across data sets, because estimates of CV (a) depend strongly on the mean of the time series, (b) increase with the length of the time series, (c) are biased when non‐normally distributed data sets are used, and (d) present a pathological behavior when rare events occur in a time series. Heath developed the proportional variability (PV) index (Heath, 2006) to try to solve these problems, defined as:

$$\text{PV}=\frac{2\sum z}{n(n‐1)}$$

where *z* is

$$z=1‐\frac{\min\left(z_{i},z_{j}\right)}{\max\left(z_{i},z_{j}\right)}$$

where “*n*” is the length of a time series, and “*z*” the individual values to calculate the pairwise comparisons. Further, we rediscovered the consecutive disparity (*D*) index (Martín‐Vide, 1986) used for decades in the field of climatology but never used in biology. *D* can be estimated as:

$$D=\frac{1}{n‐1}\sum_{i=1}^{n‐1}\left|\ln\frac{p_{i+1}}{p_{i}}\right|$$

where *p_i_* is the value of the time series at time *i* and *n* is length of the time series. To summarize, PV assesses the proportional difference between all pairs of values within a time series, while *D* assesses the proportional differences between consecutive values. Both PV and *D* overcome the mathematical problems of CV (Fernández‐Martínez et al., 2018; Heath, 2006; Heath & Borowski, 2013) and provide clear advantages over the traditional CV for comparing data sets with different statistical properties. The main benefits of PV and *D* over CV are summarized in Table 1. *D* is also particularly useful in masting studies, because it combines information about variability and temporal autocorrelation with the previous year, variability by which plant resources and populations of seed predators and dispersers should be most affected (Espelta et al., 2008, 2017; Sala et al., 2012). CV, however, is easily tractable in an analytical way because it is based on the variance and the mean of a set of values. That has allowed it to become the basis of most theories describing temporal variability.

**TABLE 1:** Summary of the mathematical and statistical benefits of the indices of proportional variability (PV) and consecutive disparity (*D*) over the coefficient of variation (CV)

	PV	*D*	CV
Equal range for comparison across data sets	Yes	No	No
Chronology of the time series matters	No	Yes	No
Robust estimates when comparing data sets with very different means	Yes	Yes	No Negatively correlated with the mean
Robust estimates when comparing data sets of different sizes	Yes	Yes	No Positively correlated with the size
Robust estimates across non‐normally distributed data sets	Yes	Yes	No Pathological increase with rare events
Detects high number of zeros as low variability (Figure 2)	Yes	Yes	No Highest values at highest percentage of zeros

Note

Properties of the indices are based on (Fernández‐Martínez et al., 2018; Heath, 2006; Heath & Borowski, 2013) and our results.

Crone et al. (2011) had already warned the masting community about the potential of CV to be a poor metric for measuring the temporal variability of reproductive efforts and that interpreting CV should be considered in context. To date, only a few studies analyzed their data sets using *D* or PV (Bogdziewicz, Fernández‐Martínez, et al., 2020; Fernández‐Martínez et al., 2017, 2019; Koenig et al., 2020; Pesendorfer et al., 2020; Vergotti et al., 2019) after our first warning about the problems linked to using CV for masting was published (Fernández‐Martínez et al., 2018). Ascari et al. (2020) still used CV but suggested that new indices should also be used. Most authors, however, have not yet been persuaded to use new metrics and have used CV without mentioning problems of comparability among different data sets. Results based on CV may still be correct, but biased results are also possible depending on the characteristics of the data sets.

**FIGURE 2:**
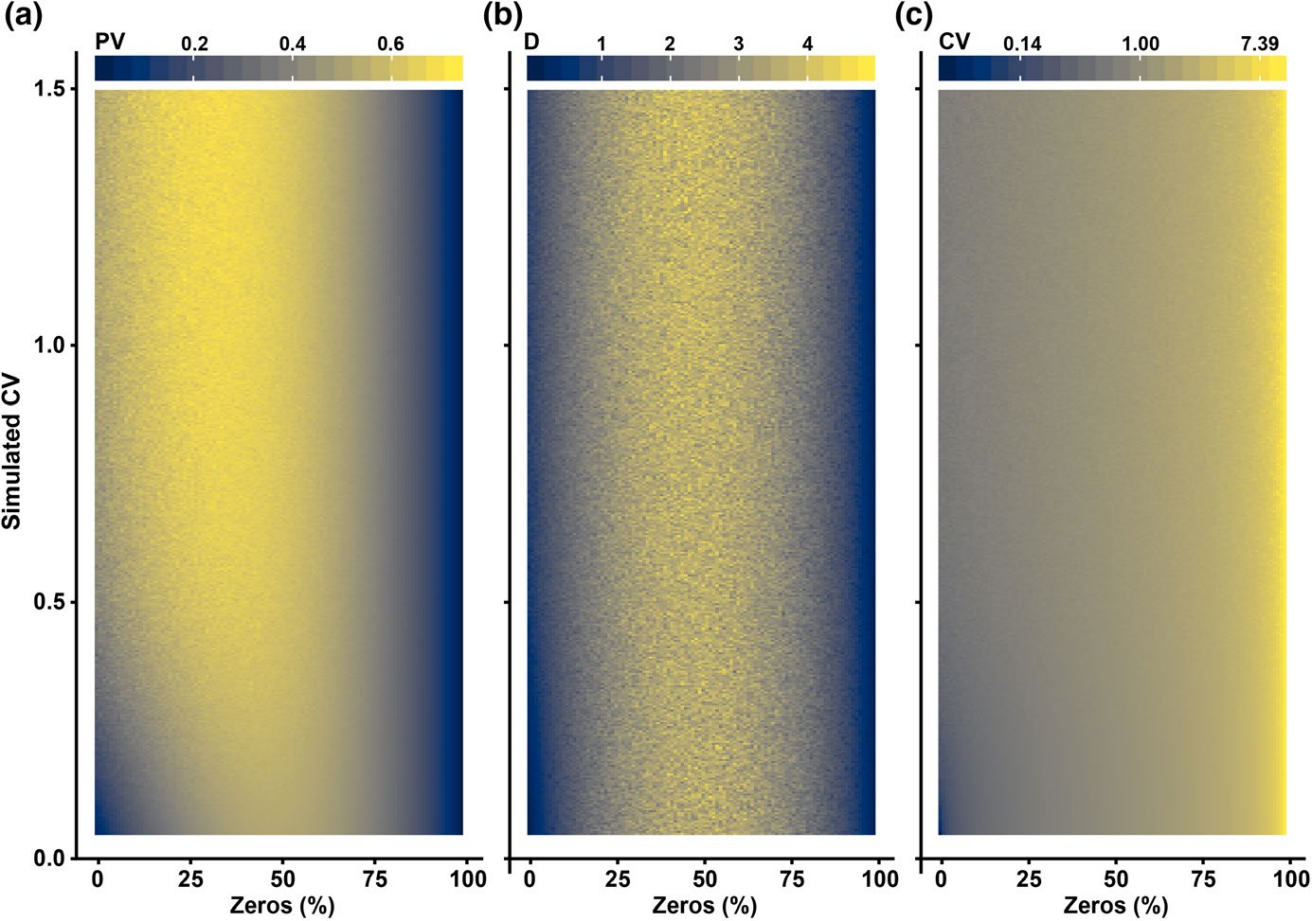
Behavior of (a) proportional variability (PV), (b) consecutive disparity (*D*), and (c) the coefficient of variation (CV) to increasing variability and the percentage of zeros in a time series. Estimates of variability using PV and *D* increase with increasing the originally simulated CV (*y*‐axis) and peak at around 50% of zeros in a time series. Instead, estimates of CV are mainly driven by the percentage of zeros, reaching the highest estimates at >90% of zeros. Notice that the color scale in panel c has been log‐transformed to try to maximize the different response of CV across the proportion of zeros and simulated variability. The code for performing the simulation is available in Supplementary Materials

Recurrent arguments for maintaining the use of CV are that CV is strongly correlated with the proportion of zeros in a time series and that *masting is about skipping reproductive attempts* (see the hypothesis of predator satiation (Espelta et al., 2008; Kelly & Sork, 2002)), for which the correlation between CV and zeros could be desirable (but implies that variability and frequency of reproduction is confounded). The statement that CV is strongly correlated with the percentage of zeros is essentially correct (Figure 2), but a time series with most of its values (e.g., 95%) *being equal* (in this case to zero) cannot be considered *highly variable*, which is one of the foundations of masting behavior so far. PV and *D* thus increase with the variability of a time series, but when time series present approximately more than 50% of zeros, they relate increasing the percentage of zeros with decreasing variability, as one would intuitively think. Most importantly, the variability of CV across data sets is dangerously driven by the percentage of zeros (i.e., frequency of reproduction) rather than by differences in variability across the data sets. That should preclude the use of an index that is supposed to measure variability, at least when the percentage of zeros is large. Hence, CV may not only not have any mathematical advantage over PV or *D* but it also has the drawback of misleadingly interpreting *a large number of equal values as high variability*. Any conclusion drawn regarding variability based on the use of CV on a data set with a large number of zeros should, therefore, be very carefully addressed.

CV, however, correlates well with PV and *D* when time series are normally distributed, and the proportion of zeros is accordingly low, and, thus, it could still be used in order to provide comparisons with previous studies. Most of the time series of highly variable reproduction in plants, though, are not normally distributed (Fernández‐Martínez et al., 2017; Kelly, 1994; Silvertown, 1980). This observation is particularly exacerbated when assessing time series of individuals instead of populations, for which the percentage of zeros can dramatically exceed 50% because of an intrinsic higher temporal variability at the individual scale (Bogdziewicz, Szymkowiak, Calama, et al., 2020). CVs would then be measuring temporal variability for individuals with a low proportion of zeros, but for individuals with a large percentage of zero values CVs would only be measuring zeros (i.e., frequency of reproduction, and not its temporal variability). These comparisons using CVs are therefore unreliable and conceptually misleading, because two different, and to some degree opposed, processes are involved. We suggest that we should separate the measurement of temporal variability, using either PV or *D* (if the chronological order of the values is important), from the frequency of reproduction, easily measured as the percentage of reproductive events over a total of observations. Both reproductive behaviors could thus be estimated for any potential data set without risking biased results nor providing misleading conclusions regarding the variability of the data sets.

## 4 SHOULD WE THUS REDEFINE MASTING?

**FIGURE 3:**
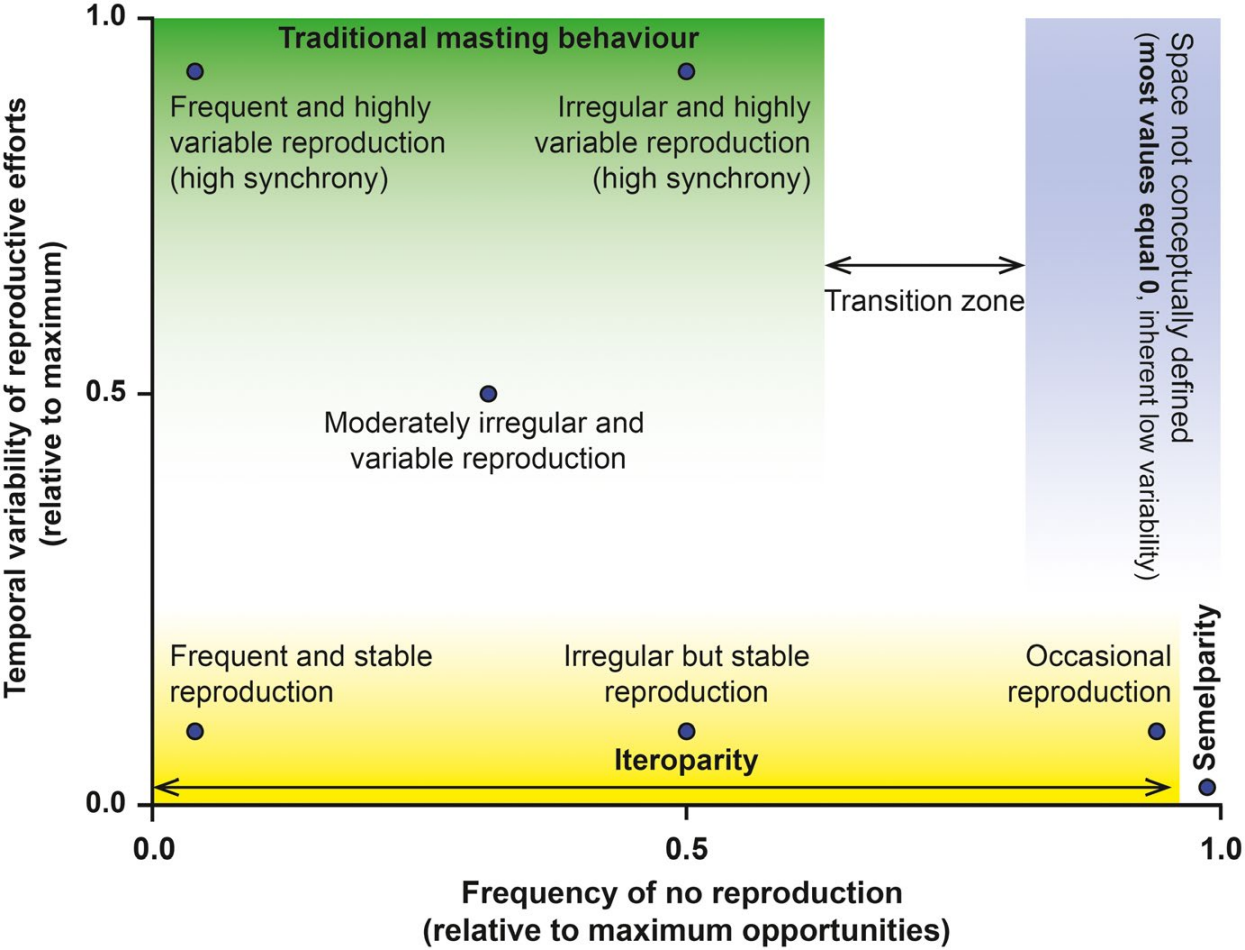
Scheme showing different types of reproductive behavior. Reproductive behavior is described as a function of the frequency of no reproduction (or percentage of zeros as in Figure 2, *x*‐axis) and the temporal variability of reproductive efforts (*y*‐axis, relative to maximum temporal variability). Traditionally defined masting behavior would be placed at high levels of temporal variability and between frequent and irregular reproduction. Notice that highly variable data sets are not possible when the frequency of no reproduction is high, because most of the values of the time series are equal (0)

We probably should. Our suggestion would lead to a scenario in which we would be able to describe the temporal behavior of reproductive efforts based on Figure 3: (a) from temporally variable to stable reproduction and (b) from frequent to occasional reproduction in iteroparous species to single reproductive event in a lifetime (in semelparous species). Full comparability could be achieved across any type of organism using PV and the frequency of reproduction because both metrics are restricted to values between 0 and 1 (Table 1). We could then even establish working thresholds to determine whether a species is likely to have masting behavior, if even deemed necessary. Some authors consider that occasional or extremely infrequent reproduction and low temporal variability equals masting (e.g., bamboo flowering once after >60 years (Kitzberger et al., 2007), individuals skipping reproduction largely more than 50% of the years (Bogdziewicz, Szymkowiak, Calama, et al., 2020; Bogdziewicz, Szymkowiak, Tanentzap, et al., 2020)), but that reproductive behavior does not match with the current definition of masting because of the mathematical concept of temporal variability. The “masting” community of researchers should definitely try to reach a consensus and redefine the traditional concept of masting, which is so far based on high temporal variability and synchrony.

From our point of view, and given the current diverse use of the term, masting is mainly a cultural and arbitrary term, referring to some species producing many seeds in some years and very few, or none, in others. Temporal variability and frequency of reproduction follow a continuous gradient across species (Fernández‐Martínez et al., 2019; Herrera et al., 1998; Kelly, 1994), so dichotomous classifications (i.e., masting species, nonmasting species) do not really make much sense. We think we should not try to classify species as masting or nonmasting but instead systematically describe their behavior by (i) the degree of temporal variability and frequency of reproduction, (ii) the synchronicity of reproduction among individuals, and (iii) the degree of dependence between reproduction in consecutive years. We, therefore, recommend avoiding the use of the term “masting” in the scientific literature due to the confusion that it causes and use instead terms defining what we are actually studying: variable plant reproduction. Avoiding the use of an ambiguous term such as masting will help the scientific community to describe reproductive behaviors using purely mathematical and universally comparable terms such as temporal variability and frequency.

## CONFLICT OF INTEREST

The authors declare no conflict of interests.

## AUTHOR CONTRIBUTIONS

**Marcos Fernández‐Martínez:** Conceptualization (lead); Formal analysis (lead); Funding acquisition (equal); Investigation (equal); Methodology (lead); Writing‐original draft (lead); Writing‐review & editing (equal). **Josep Peñuelas:** Conceptualization (supporting); Funding acquisition (equal); Investigation (equal); Methodology (equal); Writing‐original draft (supporting); Writing‐review & editing (equal).

## Supporting information

Supplementary materials

## Data Availability

Data sharing is not applicable to this article as no new data were created or analyzed in this study. The code for performing all simulations is available in Supplementary Materials.
