# Supplementary material for: Measuring temporal patterns in ecology: The case of mast seeding
Source: Ecol Evol. 2021 Mar 11;11(7):2990–6. doi: 10.1002/ece3.7291 (PMC8019024; doi:10.1002/ece3.7291)
Supplement: Supplementary file 1 — Supplementary materials [file ECE3-11-2990-s001.pdf]

Does masting need new metrics and a new definition?
Supplementary materials

Simulation for Figure 1

These results are used in Figure 1 and in the main text

Population synchrony vs. temporal variability

```
# 3 populations of 100 individuals with increasing variability
# and correlation between individuals
synlist <- list()
syndat <- matrix(nrow = 100, ncol=500)
sdcors <- expand.grid(sds=c(0.1, 1, 10),
                      cors=seq(0.0, 0.99, length=50))
synres <- data.frame(sd=sdcors$sds, pv=NA, d=NA, cv=NA,
                     sdcor=sdcors$cors, cor=NA)

# calculations start here
set.seed(1)
for (i in 1:nrow(synres)){
  mu <- rep(0,100)
  Sigma <- matrix(sdcors$cors[i], nrow=100, ncol=100)
  diag(Sigma) <- 1
  rawvars <- mvrnorm(n=100, mu=mu, Sigma=Sigma)
  syndat <- qgamma(pnorm(rawvars), sdcors$sds[i], scale=100)
  synlist [[i]] <- syndat
    synres [i,"pv"] <- pv.fun(rowMeans(syndat))
    synres [i,"d"] <- d.fun(rowMeans(syndat))
    synres [i,"cv"] <- cv.fun(rowMeans(syndat))
    mat <- round(cor(syndat), 2)
    diag(mat)<-NA
    synres [i,"cor"] <- mean(mat, na.rm=T)
}

# Additional graphs - CV
ggplot(synres, aes(cor, cv, fill= as.factor(sd))) +
  ylab("Temporal variability (CV)") +
  xlab("Population synchrony (r)") +
  geom_point(shape=21, color="black", size=3) +
  geom_smooth(se = T, method = loess) +
  theme_cowplot(font_size = 20, line_size = 1.25) +
  theme(legend.background = NULL,
        legend.direction = "horizontal",
```

```
        legend.position = "top")
```

```
## `geom_smooth()` using formula 'y ~ x'
```

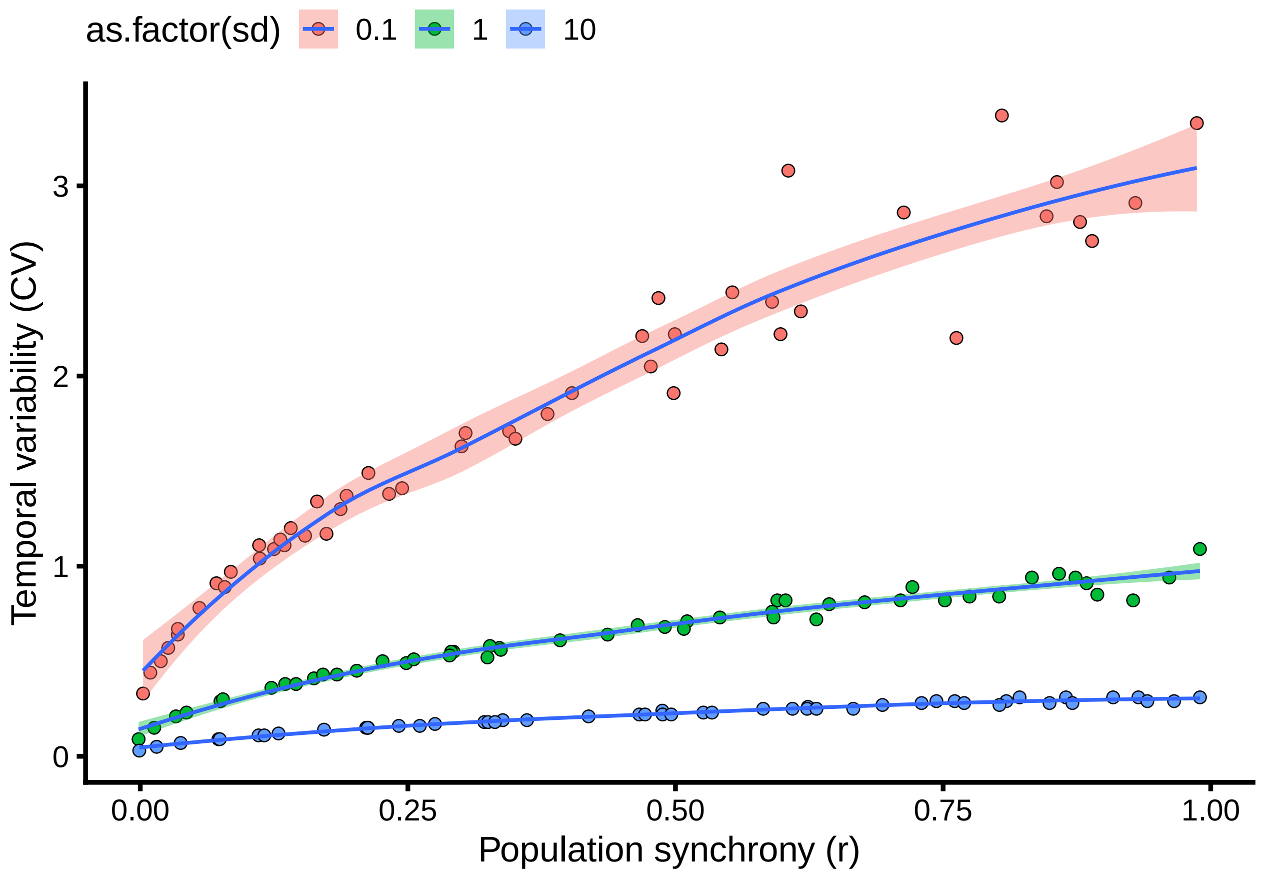


```
# Additional graphs - D
ggplot(synres, aes(cor, d, fill= as.factor(sd))) +
  ylab("Temporal variability (D)") +
  xlab("Population synchrony (r)") +
  geom_point(shape=21, color="black", size=3) +
  geom_smooth(se = T, method = loess) +
  theme_cowplot(font_size = 20, line_size = 1.25) +
  theme(legend.background = NULL,
        legend.direction = "horizontal",
        legend.position = "top")
```

```
## `geom_smooth()` using formula 'y ~ x'
```

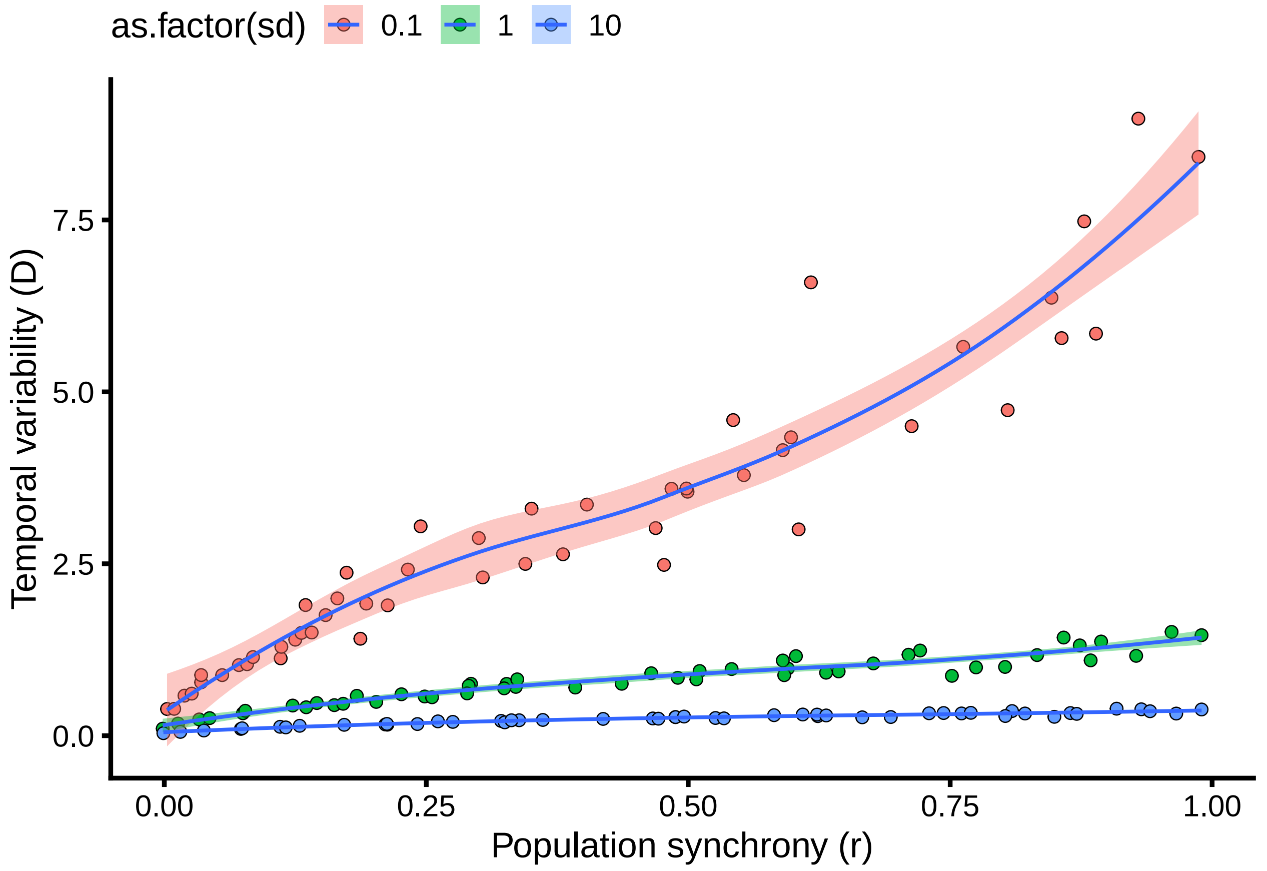

** Figure 1 **

```
ggplot(synres, aes(cor, pv, fill= as.factor(sd))) +
  ylim(0,0.96) + ylab("Temporal variability (PV)") +
  xlab("Population synchrony (r)") +
  geom_point(shape=21, color="black", size=3) +
  geom_smooth(se = T, method = loess) +
  theme_cowplot(font_size = 20, line_size = 1.25) +
  theme(legend.background = NULL,
        legend.direction = "horizontal",
        legend.position = "top")
```

```
## `geom_smooth()` using formula 'y ~ x'
```

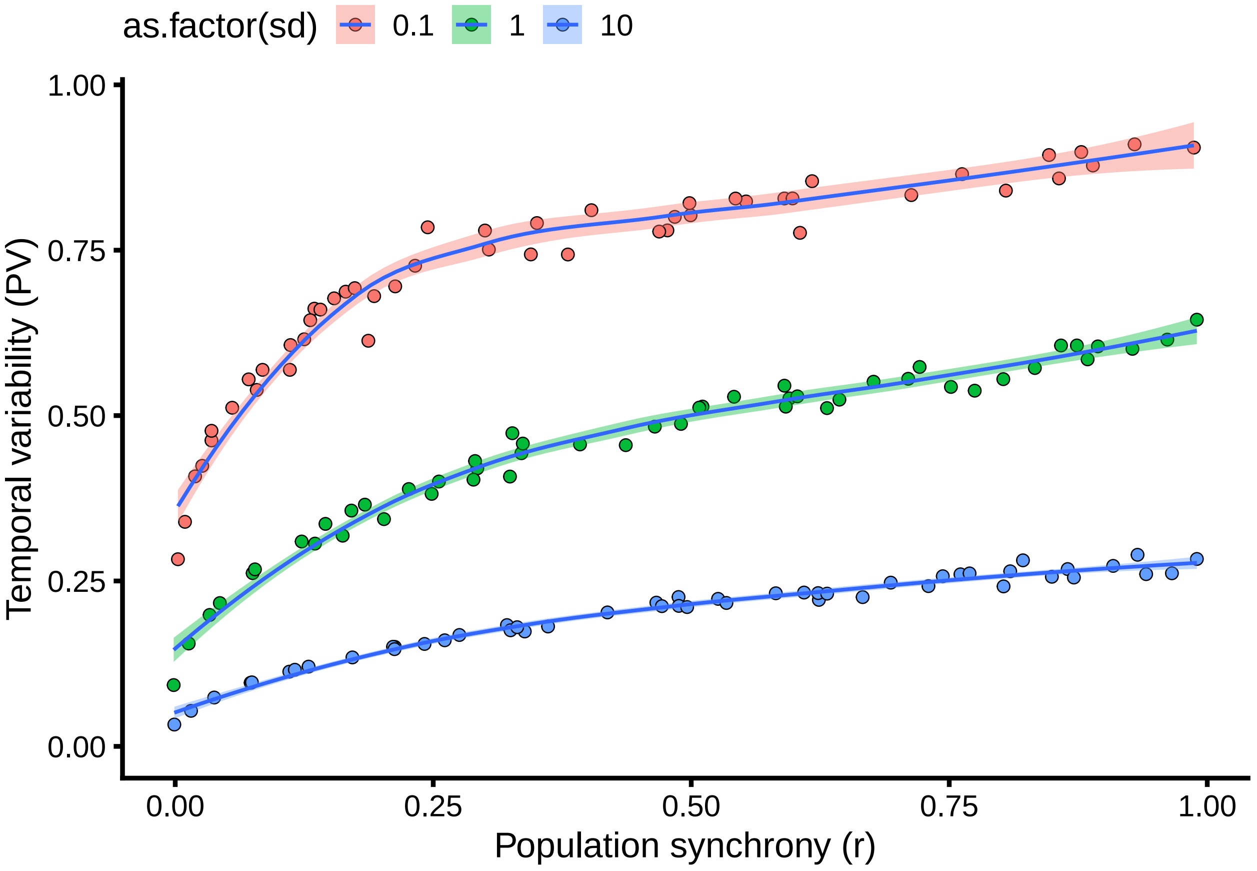

Simulation for Figure 2

These results are used in Figure 2 and in the main text

Simulated variability (CV) and % of zeros vs PV, D and CV

```
# Simulation mean = 1000
meann <- 1000 # mean of 1000 variables
var.max <- 1.5 # proportional to the mean
var.min <- 0.05 # proportional to the mean
runs <- 200
a <- c(1:100) #  from 1 to 100 to select rows to be 0
sds.zers <- expand.grid(sds=seq(meann*(var.min),
                                meann*(var.max), by=4),
                        zers=seq(0, 99, by=1))
res <- data.frame(pvs=NA, cvs=NA, ds=NA, semiter=NA,
                  geom.mean=NA, freq=NA, zeroes=sds.zers$zers,
                  sd=sds.zers$sds, real.sd=NA)
data <- matrix(nrow=100, ncol=nrow(res))

set.seed(1)
# calculations start here
for (i in 1:nrow(res)){
  data [,i] <- rnorm(n=100, mean=meann,
                         sd=res$sd[i])
  # check for negative values in data #
```

```
  data [which(data [,i]<0),i] <- runif(length(which(data [,i]<0)),
                                       min=1, max=max(data [,i], na.rm=T))
  nums <- sample(a, size=res$zeroes[i], replace=F) # all these random rows are 0
  data [nums,i] <- 0
  res$pvs[i] <- pv.fun(data[,i])
  res$cvs[i] <- cv.fun(data[,i])
  res$ds[i] <- d.fun(data[,i], k=1)
  res$real.sd[i] <- sd(data[,i])
}

# Heatmap
pv1000 <- ggplot(res, aes(zeroes, (sd/1000), fill= pvs)) +
  geom_raster(interpolate=T, hjust = 0, vjust = 0) +
  scale_fill_viridis(name = "PV", option = "E") +
  theme_cowplot() +
  xlab ("Zeros (%)") + ylab("Simulated CV (SD/Mean)") +
  theme(legend.position = "top")
d1000 <- ggplot(res, aes(zeroes, (sd/1000), fill= ds)) +
  geom_raster(interpolate=T, hjust = 0, vjust = 0) +
  scale_fill_viridis(name = "D", option = "E") +
  theme_cowplot()  +
  xlab ("Zeros (%)") + ylab(NULL) +
  theme(legend.position = "top")
cv1000 <- ggplot(res, aes(zeroes, (sd/1000), fill= cvs)) +
  geom_raster(interpolate=T, hjust = 0, vjust = 0) +
  scale_fill_viridis(name = "CV", option = "E", trans = "rndlog") +
  theme_cowplot() + # add , trans = "log" in scale_fill_viridis?
  xlab ("Zeros (%)") + ylab(NULL) +
  theme(legend.position = "top")

plot_grid(pv1000, d1000, cv1000, ncol=3, nrow=1,
          align="hv", labels="auto", rel_widths = c(1, 0.90, 0.9),
          rel_heights = c(1, 1, 1), axis = "rb")
```

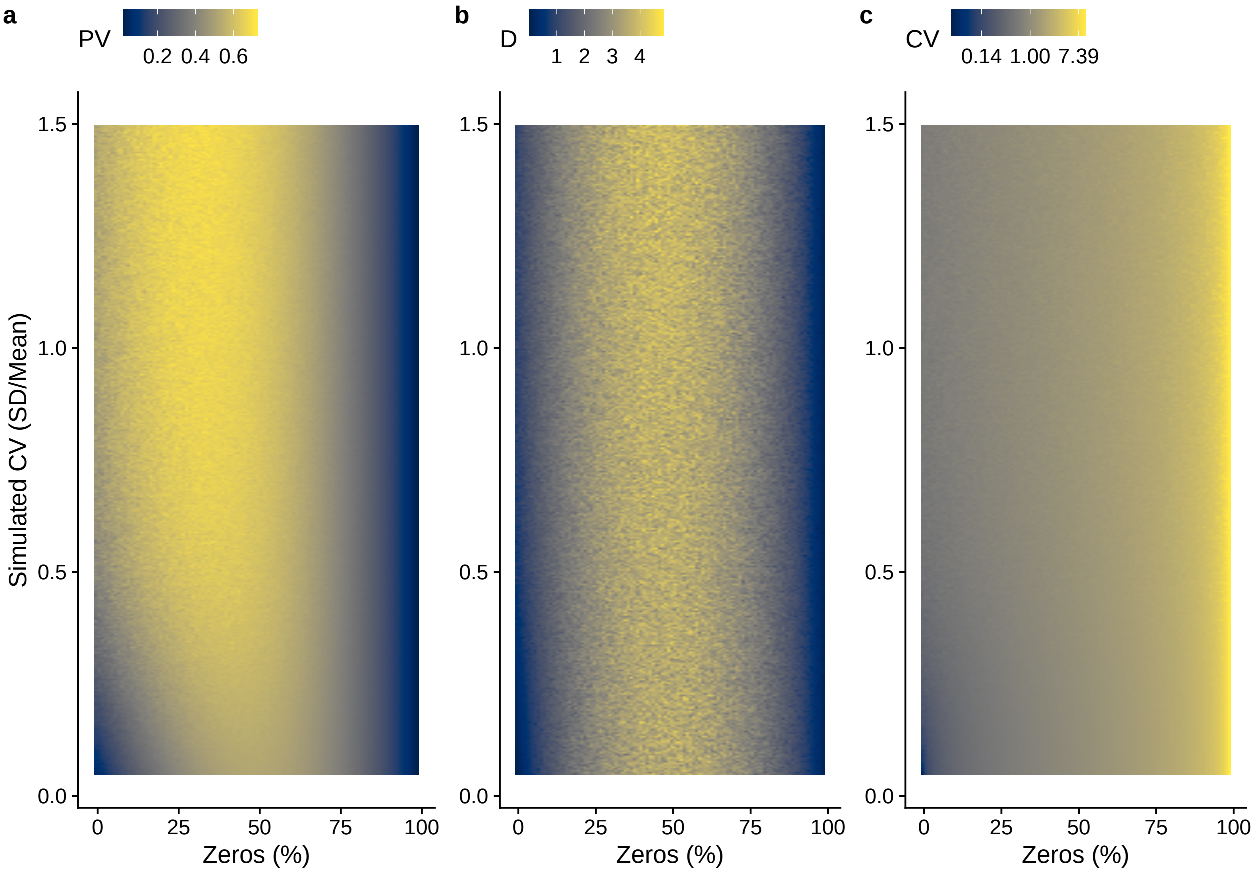
a
PV
0.2 0.4 0.6
b
D
1 2 3 4
c
CV
0.14 1.00 7.39
Simulated CV (SD/Mean)
0.0
0.5
1.0
1.5
0 25 50 75 100
Zeros (%)
